# Solvothermal synthesis of Fe_3_O_4_ nanospheres for high-performance electrochemical non-enzymatic glucose sensor

**DOI:** 10.1038/s41598-020-73090-4

**Published:** 2020-09-29

**Authors:** Jiasheng Xu, Yuting Sun, Jie Zhang

**Affiliations:** 1 College of Chemistry, Chemical Engineering and Environmental Engineering, Liaoning Shihua University, Fushun, 113001 People’s Republic of China; 2 Liaoning Province Key Laboratory for Synthesis and Application of Functional Compounds, College of Chemistry and Chemical Engineering, Bohai University, Jinzhou, 121013 People’s Republic of China

**Keywords:** Sensors and biosensors, Electrocatalysis

## Abstract

Ferroferric oxide (Fe_3_O_4_) nanospheres have been synthesized via a facile solvothermal procedure to serve as an electrode material for high performance non-enzymatic glucose sensor. The as-synthesized Fe_3_O_4_ nanospheres with a uniform size from 16 to 18 nm, which can increase the reaction contact area and the active sites in the process of glucose detection. Benefiting from the particular nanoscale structure, the Fe_3_O_4_ nanospheres obviously enhanced the activity of electrocatalytic oxidation towards glucose. When the Fe_3_O_4_ nanospheres material was used for non-enzymatic glucose sensor, several electrochemical properties including the high sensitivity 6560 μA mM^−1^ cm^−2^ (0.1–1.1 mM), limit of detection 33 μM (S/N = 3) and good long-term stability were well demonstrated. Furthermore, Fe_3_O_4_ nanospheres electrode confirmed the excellent performance of selectivity in glucose detection with the interfering substances existed such as urea, citric acid, ascorbic acid, and NaCl. Due to the excellent electrocatalytic activity in alkaline solution, the Fe_3_O_4_ nanospheres material can be considered as a promising candidate in blood glucose monitoring.

## Introduction

Glucose is the predominant energy-producing substance for metabolism of human body and the levels of the blood glucose must be stable in a certain range to maintain the body activities^1–4^. However, high levels of the blood glucose that existed in human body may lead to diabetes and complications^5^. Diabetes, a serious metabolic disease, has become a global health problem as a growing threat to human body^6–9^. Hence, the use of appropriate treatment to monitor and detect glucose concentration in human blood has been particularly significant^10–13^. Several common techniques such as optical^14^, acoustic^15,16^, fluorescent^17^ and electrochemical method^18^ have been utilized for glucose monitoring and detection. Enzymatic glucose sensor and non-enzymatic glucose sensor are the earliest biosensor studied by researchers in the field of glucose analysis^19^. Enzymatic glucose sensor is usually based on the catalytic performance of glucose oxidase (GOx) to achieve the specific detection of glucose. Researchers have made a series of advances on enzymatic glucose sensor. Ramanavicius et al.^20^ evaluated the impedimetric glucose sensor based on the electrodes modified by both 1,10-Phenanthroline-5,6-dione and glucose oxidase. Valiuniene et al.^21^ investigated the glucose biosensor based on graphite electrode modified by Prussian blue, polypyrrole and glucose oxidase. Up to now, for some shortcomings including high-cost, low lifetime and poor stability, enzymatic electrochemical glucose sensor has been gradually replaced by non-enzymatic electrochemical glucose sensor^22^. Electrochemical non-enzymatic glucose sensor has played a key role in clinical diagnosis^23^.

In recent years, due to the high accuracy, sensitivity and efficiency, the electrochemical non-enzymatic glucose sensor has become a leading technology with boundless potential^24–26^. As the electrode material, noble metals are limited by the high cost. A series of transition metal oxides have become common materials for constructing electrochemical non-enzymatic glucose sensors^27^. Some transition metal oxides such as MnO_2_, CuO, Co_3_O_4_ and NiO were extensively studied because all of them could be utilized as alternatives to noble metals^28^. Nanomaterials have gradually become the most popular research direction due to their various excellent properties, which also leads to a new level of research in the field of sensing^29^. Due to low toxicity, biocompatibility, superparamagnetism and catalytic activity, Fe_3_O_4_ nanomaterials have received considerable interest^30–32^. Aside from the inherent properties, the structure and morphology of the Fe_3_O_4_ nanomaterials are also the key factors affecting the electrochemical sensing performance in glucose detecting^33^. It has been reported that the Fe_3_O_4_ nanoparticles in various shapes synthesized via different methods possess different properties^34,35^. With the kinetic capacity in glucose oxidation reaction and the large surface area, nano-spherical structures can offer more active sites and enhance the electrochemical performance^36–38^. Therefore, a facile process to synthesize Fe_3_O_4_ nanomaterials as the material of electrochemical non-enzymatic glucose sensor has become essential.

In this paper, a high-performance electrochemical non-enzymatic glucose sensor has been presented. The Fe_3_O_4_ nanospheres have been synthesized in a facile solvothermal procedure, which are pasted on the Ni foam. Several electron microscopic analysis and diffraction techniques were used to characterize the properties of microstructure and morphology of Fe_3_O_4_ nanospheres. Satisfactorily, the Fe_3_O_4_ nanospheres displayed superior nanoscale size and electrocatalytic properties for detecting glucose (from 0.1 to 1.1 mM) and a limit of detection is 33 μM.

## Results and discussion

**Figure 1:**
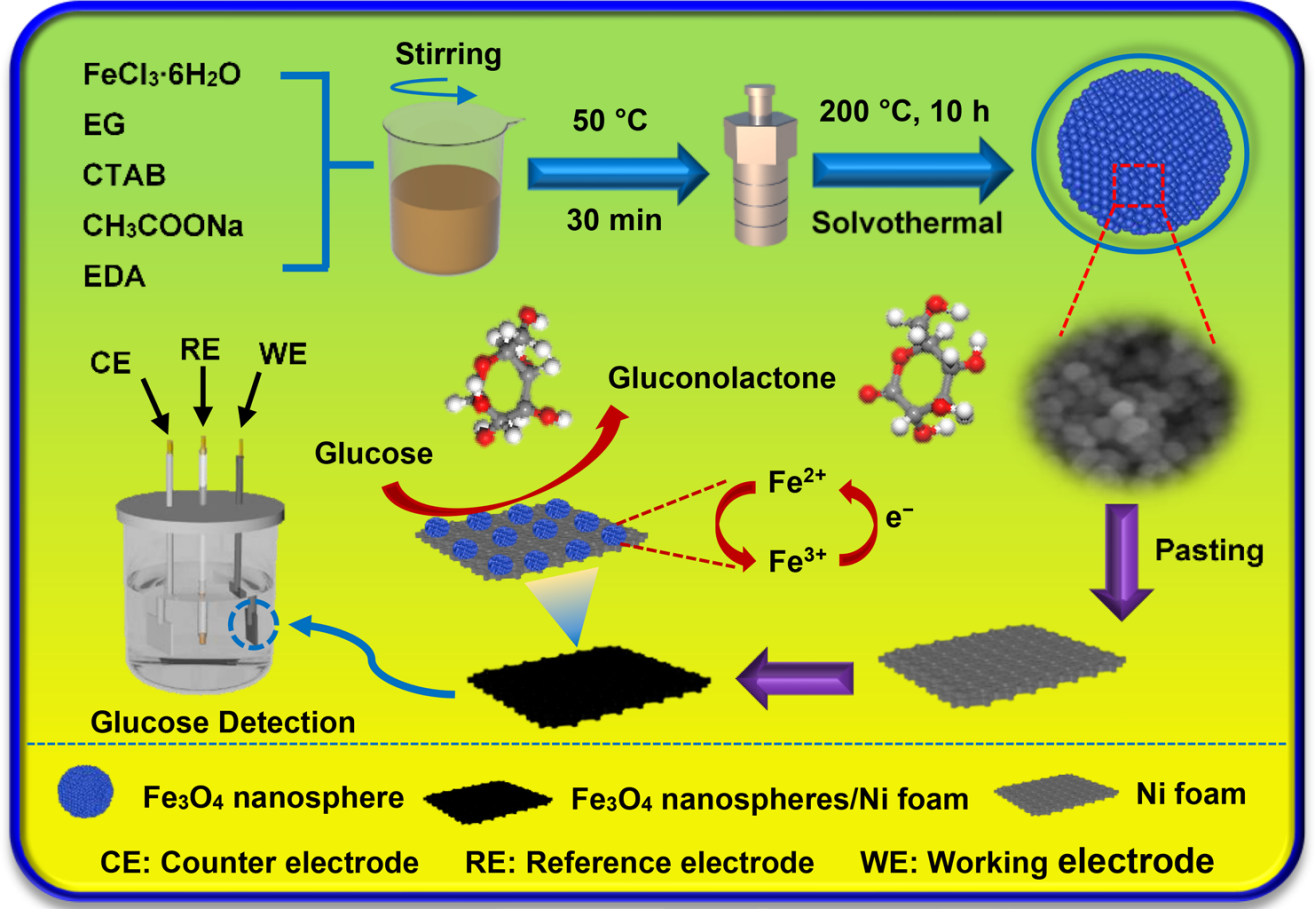
The schematic illustrations of the synthesis and electrochemical glucose detection process of the Fe_3_O_4_ nanospheres electrode.

The representative synthesis and electrochemical glucose detection process of the Fe_3_O_4_ nanospheres electrode is exhibited in Fig. 1. The synthesis of Fe_3_O_4_ nanospheres was carried out in a solvothermal procedure and the as-synthesized Fe_3_O_4_ nanospheres electrode was acted as a working electrode in three-electrode system for glucose detection. Firstly, FeCl_3_·6H_2_O, CTAB (hexadecyl trimethyl ammonium bromide), CH_3_COONa and EDA (ethylenediamine) were dissolved in EG (ethylene glycol) under magnetic stirring for 30 min at 50 °C. Then the mixed reagents were transferred to the autoclave to synthesize the target samples. The as-synthesized Fe_3_O_4_ nanospheres samples were pasted on Ni foam for electrochemical detecting. In the process of electrochemical sensing towards glucose, the glucose was oxidized into gluconolactone by the strongly oxidized Fe^3+^ which was formed rapidly in the NaOH electrolyte. At the same time, the electron transport reactions also occurred. Subsequently, the electrons were transferred to the Ni foam. The electrocatalytic oxidation towards glucose was finally realized under the interaction in the three-electrode system.

**Figure 2:**
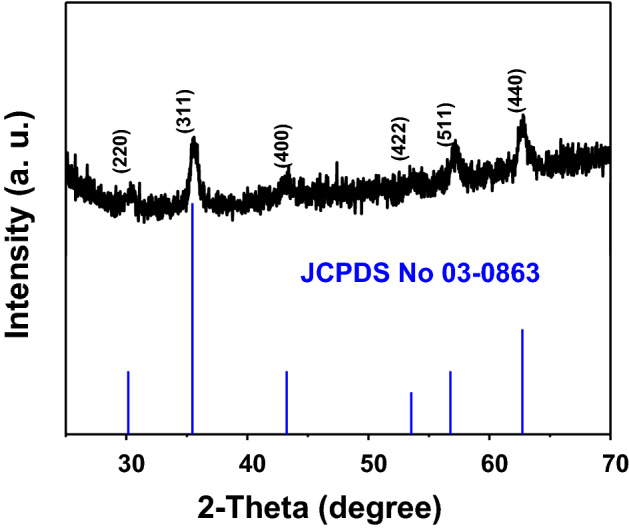
XRD patterns of the Fe_3_O_4_ nanospheres synthesized by solvothermal reaction at 200 °C for 10 h. The vertical lines at bottom are the standard diffraction peaks of Fe_3_O_4_ from JCPDS card No. 03-0863.

Crystallographic data and chemical composition of Fe_3_O_4_ nanospheres were identified by the powder X-ray diffraction analysis (XRD, in the 2*θ* range from 25° to 70°). All characteristic peaks are well consistent with the standard PDF card (JCPDS No. 03-0863) in Fig. 2, which indicate the products are pure Fe_3_O_4_. All characteristic peaks at 30.3°, 35.4°, 43.4°, 53.5°, 56.9° and 62.6° represent the (220), (311), (400), (422), (511) and (440) crystal faces of Fe_3_O_4_, respectively.

**Figure 3:**
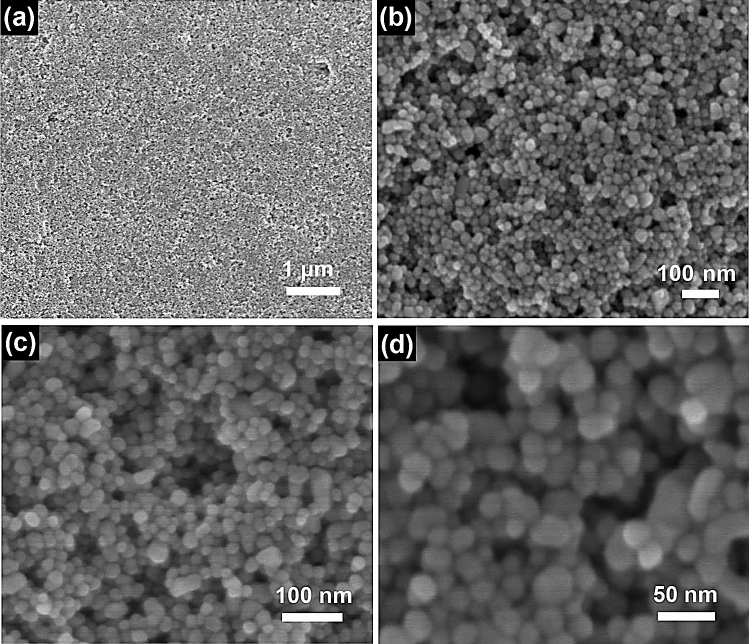
SEM images of Fe_3_O_4_ nanospheres sample. (**a**,**b**) Low magnification SEM images of Fe_3_O_4_ nanospheres. (**c**,**d**) High magnification SEM images of Fe_3_O_4_ nanospheres.

In order to observe surface of the samples, the morphologies of Fe_3_O_4_ nanospheres has been presented by scanning electron microscope (SEM). The low magnification SEM microstructure images (in Fig. 3a,b) present an overall morphology of Fe_3_O_4_ nanospheres. The products with extremely small size exhibit a fine exterior surface and gathered together. Figure 3c,d show the high magnification SEM microstructure images of Fe_3_O_4_ nanospheres. It is observed that every Fe_3_O_4_ nanosphere is in general of sphere-like and possesses a uniform size of 16–18 nm.

**Figure 4:**
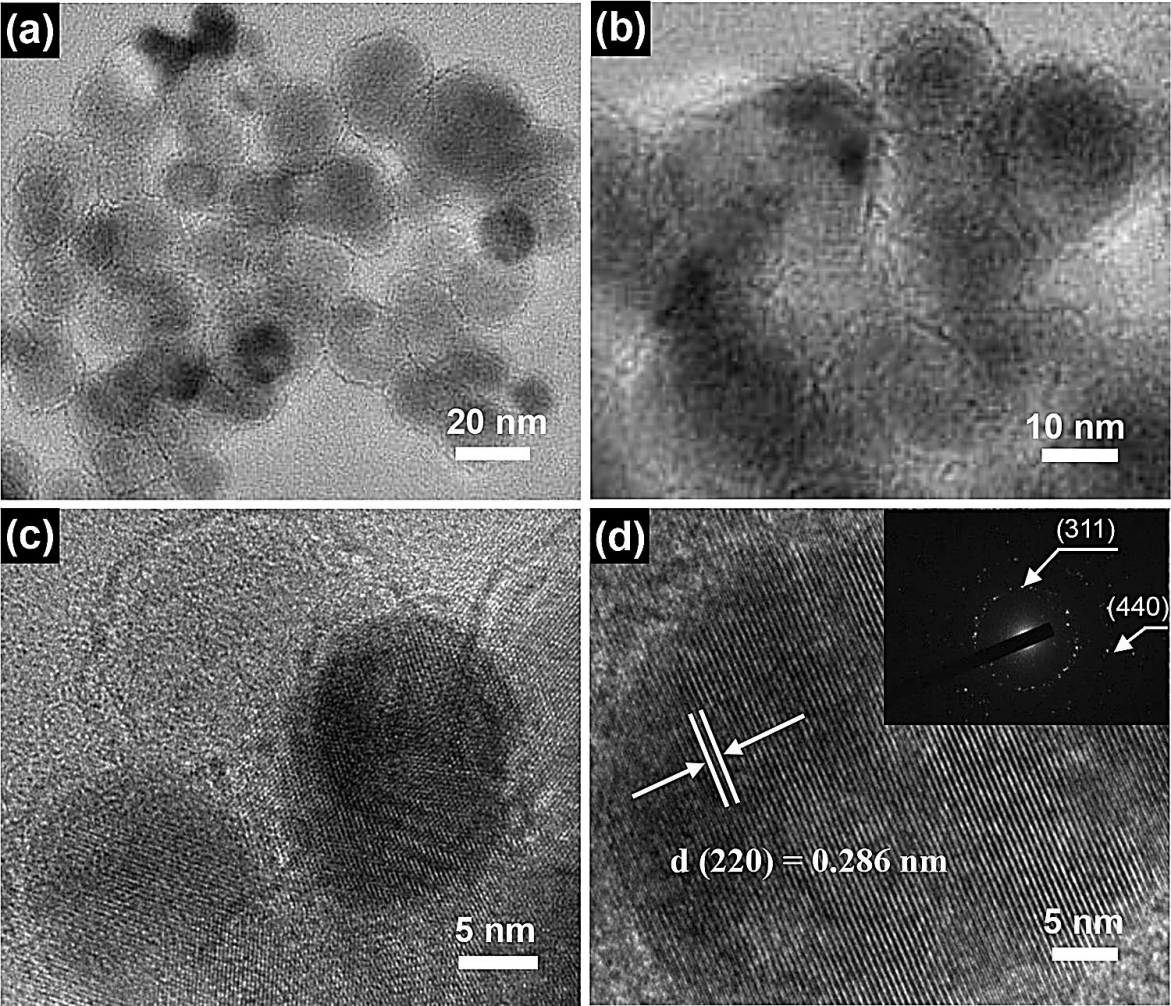
TEM images of Fe_3_O_4_ nanospheres sample. (**a**,**b**) TEM images of Fe_3_O_4_ nanospheres. (**c**,**d**) HRTEM images of Fe_3_O_4_ nanospheres; inset is the SAED pattern of Fe_3_O_4_ nanospheres.

The clearer structural properties of the as-synthesized Fe_3_O_4_ nanospheres were further presented by transmission electron microscope (TEM). Figure 4a,b display the typical TEM images of as-synthesized Fe_3_O_4_ nanospheres with a large quantity of well-dispersed nanospheres. Figure 4c,d display the high-resolution TEM (HRTEM) images with a clear lattice structure property. The spacing of lattice is calculated to be 0.286 nm, which is consistent with (220) interplanar spacing of the Fe_3_O_4_ nanospheres. Inset is the SAED pattern, the diameters of the two planes correspond to the (311) and (440) planes, which indicates that the Fe_3_O_4_ nanospheres have an excellent crystalline structure.

**Figure 5:**
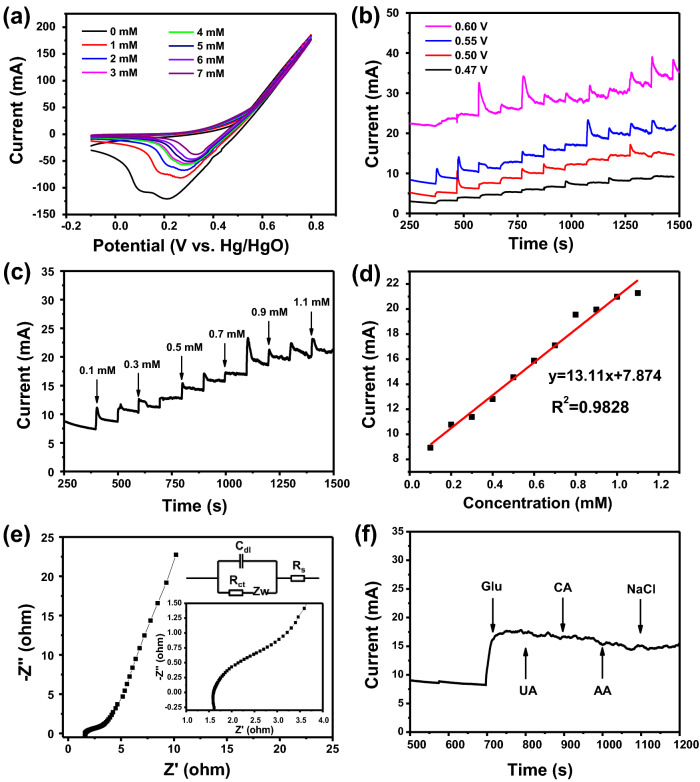
(**a**) CV curves of Fe_3_O_4_ nanospheres electrode in 0.5 M NaOH containing various concentration of glucose (0–7 mM) at a scan rate of 20 mV s^−1^. (**b**) Effect of different potentials on amperometric response of Fe_3_O_4_ nanospheres electrode to the successive addition of 0.1 mM glucose. (**c**) Amperometric response of Fe_3_O_4_ nanospheres electrode upon the addition of various concentrations of glucose at 0.55 V. (**d**) Corresponding calibration curve of the response current density and glucose concentration. (**e**) Nyquist plot of the Fe_3_O_4_ nanospheres electrode. Inset is the equivalent circuit. (**f**) Amperometric response of the Fe_3_O_4_ nanospheres electrode towards the addition of 1 mM glucose and 0.1 mM different interfering substances.

The CV curves of Fe_3_O_4_ nanospheres electrode under various glucose concentration (0–7 mM) in 0.5 M NaOH are displayed in Fig. 5a. It is obvious from these CV curves that the cathodic peak potential shows a slight positive shift when glucose concentration is increased gradually. The observation reveals that the Fe_3_O_4_ nanospheres electrode possesses a great advantage in electrocatalytic activity. In the glucose detecting process, some values such as applied working potential has played an important role. Figure 5b shows the current–time curves of Fe_3_O_4_ nanospheres electrode when adding glucose successively for every 100 s at various applied potential in 0.5 M NaOH. By comparison, Fe_3_O_4_ nanospheres electrode displays a weak sensitivity at the highest applied potential 0.60 V and the noise interference is large. Hence, the appropriate potential value option for Fe_3_O_4_ nanospheres electrode is 0.55 V. The ability of the Fe_3_O_4_ nanospheres electrode towards glucose electro-oxidation was further investigated at the selected potential of 0.55 V. As described in Fig. 5c, amperometric response increased significantly with the increase of glucose concentration in the NaOH. This result reveals an excellent electrocatalytic ability of Fe_3_O_4_ nanospheres electrode for detecting glucose. The calibration curve of the current density and glucose concentration are displayed in Fig. 5d, which determined the relevant measurement range of 0.1–1.1 mM (R^2^ = 0.9828). The sensitivity of Fe_3_O_4_ nanospheres electrode is 6560 μA mM^−1^ cm^−2^ by observing corresponding slope parameter and a corresponding detection limit is 33 μM (S/N = 3). We have compared sensitivity of Fe_3_O_4_ nanospheres electrode in electrochemical sensing towards glucose with the previously reported data on iron materials and other types of electrochemical sensors. Corresponding comparison of Fe_3_O_4_ nanospheres electrode with other electrode materials was summarized in Table 1. Sanaeifar et al. synthesized GOx/PVA-Fe_3_O_4_/Sn electrode and the sensitivity was 9.36 μA mM^−1^ cm^−2^; Vennila et al. prepared Ni-Co/Fe_3_O_4_/GCE which showed a sensitivity for 2171 μA mM^−1^ cm^−2^; Cao and his team synthesized Fe_2_O_3_ nanowire arrays which possessed a sensitivity for 726.9 μA mM^−1^ cm^−2^; Zhang et al. prepared 1 D Fe_3_O_4_ nanorod arrays and the sensitivity was 406.9 μA mM^−1^ cm^−2^. Obviously, compared with earlier reports, Fe_3_O_4_ nanospheres electrode has a higher sensitivity. It may benefit from the synergistic interactions between Fe_3_O_4_ nanospheres and Ni foam^39^. Electrochemical impedance spectroscopy (EIS) technique is also important to evaluate the electrochemical glucose sensor, which has been also carried out in other papers^40^. Figure 5e exhibits the Nyquist plot of Fe_3_O_4_ nanospheres electrode in the three-electrode system. Inset is a part of the Nyquist plot in the high frequency region which shows a semicircle shape with small diameter. It possesses a certain relationship with the controlled process of the charge transfer^41^. The diameter of the semicircle in Nyquist plot is equal to the charge transfer resistance (Rct) of the active surface area of the Fe_3_O_4_ nanospheres electrode, which was calculated to be 1.09 Ω. The low electrochemical impedance indicates a fast glucose oxidation kinetics in the process of glucose detection. The above series of results are sufficient to prove that the non-enzymatic glucose sensor based on the Fe_3_O_4_ nanospheres electrode possesses the excellent performance of providing the effective electron transport pathway for glucose detection. Some interfering substances, such as citric acid (CA), urea (UA) and Cl^−^ from NaCl present in the human blood may have an effect on the Fe_3_O_4_ nanospheres electrode. Here, we study the anti-interference performance of the Fe_3_O_4_ nanospheres electrode through the current–time curve. Figure 5f shows the current–time curve of Fe_3_O_4_ nanospheres electrode in NaOH solution with the presence of 1 mM glucose, 0.1 mM CA, UA, ascorbic acid (AA) and NaCl. There was a significant current response when 1 mM of glucose was in NaOH solution. On the contrary, current response had little change with 0.1 mM other interfering substances in NaOH solution, revealing that Fe_3_O_4_ nanospheres electrode has great selectivity for glucose detection.

**Table 1:** Comparison of Fe_3_O_4_ nanospheres electrode with previously reported data in the literature.

No	Electrode material	Sensitivity (μA mM^−1^ cm^−1^)	Linear range (mM)	References
1.	Gox/PVA-Fe_3_O_4_/Sn	9.36	0.005–30	^42^
2.	Ni–Co/Fe_3_O_4_	2171	0.001–11	^43^
3.	Fe_2_O_3_ nanowire array	726.9	0.015–8	^44^
4.	1 D Fe_3_O_4_ NRA	406.9	0.0005–3.67	^45^
5.	Fe_3_O_4_ nanospheres	6560	0.1–1.1	This work

**Figure 6:**
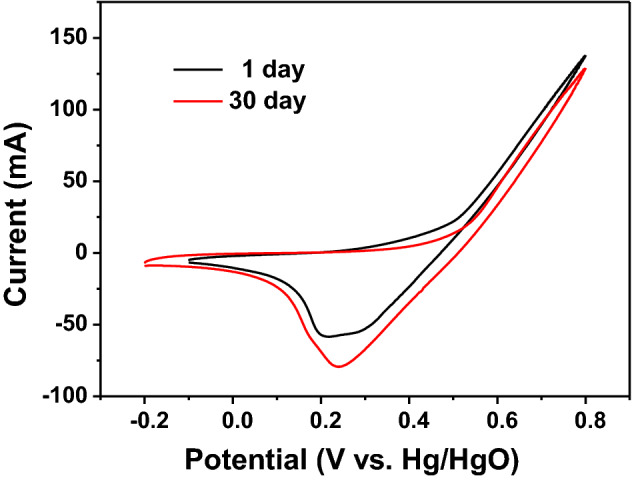
Comparing CV curves of Fe_3_O_4_ nanospheres electrode after 1 day and 30 days in 0.5 M NaOH.

Figure 6 shows the CV curves of Fe_3_O_4_ nanospheres electrode after 1 day and 30 days in 0.5 M NaOH. As can be seen from the figure, there is no obvious change in the shape of CV curve after 30 days. Furthermore, the value of the current response after 30 days did not change much, which confirmed the satisfactory stability of the Fe_3_O_4_ nanospheres electrode.

## Conclusions

In summary, Fe_3_O_4_ nanospheres with nanoscale size have been successfully synthesized in a facile solvothermal procedure. The electrochemical sensing performance of glucose in the three-electrode system has been investigated. The as-synthesized Fe_3_O_4_ nanospheres electrode demonstrated a high efficiency and excellent selectivity in electrochemical sensing of glucose. Inspiringly, in relevant measurement range (from 0.1 to 1.1 mM), Fe_3_O_4_ nanospheres electrode possesses a satisfactory sensitivity for 6560 μA mM^−1^ cm^−2^. The value of detection limit is 33 μM (S/N = 3). Thus, Fe_3_O_4_ nanospheres material has the potential to become a functional electrode material for detecting glucose in the research field of electrochemistry.

## Experimental details

### Synthesis of the Fe_3_O_4_ nanospheres electrode

All chemicals were of analytical grade. In a conventional procedure, 1.0 g ferric chloride hexahydrate (FeCl_3_·6H_2_O), 1.0 g hexadecyl trimethyl ammonium bromide (CTAB) and 3.0 g sodium acetate trihydrate (CH_3_COONa) were first added into 20 mL ethylene glycol (EG) and magnetically stirred for 20 min at 50 °C to form a uniform yellow solution. Subsequently, adding 10 mL ethylenediamine (EDA) to above-mentioned yellow mixture with magnetically stirring for 10 min. Then, the as-synthesized sample was added to a Teflon-lined autoclave (50 mL) and kept it for 10 h at 200 °C. Fe_3_O_4_ nanospheres were collected by a magnet after cooling down the room temperature, using ethanol and deionized water to rinse them repeatedly. The synthesized Fe_3_O_4_ nanospheres were mixed with acetylene black, poly (vinylidene fluoride) (PVDF) and N-methyl-2-pyrrolidone (NMP) in a good percentage. Finally, pasting above mixture on Ni foam (length × width = 20 mm × 10 mm) as the working electrode.

### Characterizations

The X-ray diffraction (XRD) was conducted on Rigaku RAD-3C diffractometer. Scanning electron microscope (SEM, JEOL S-4800), transmission electron microscope (TEM, JEOL JEM-2100F) were utilized to test the structure and morphologies of Fe_3_O_4_ nanospheres. Using an electrochemical workstation (CHI660D) for cyclic votammetry (CV) measurement and current–time analysis. Using platinum sheet for counter electrode, Hg/HgO electrode for reference electrode.
